# Supplementary material for: Splice-Junction-Based Mapping of Alternative Isoforms in the Human Proteome
Source: Cell Rep. Author manuscript; Available in PMC 2020 Jan 15. (PMC6961840; doi:10.1016/j.celrep.2019.11.026)
Supplement: 2 [file NIHMS1546469-supplement-2.zip › DF1/PXD010154/Liver/Liver_25_UBE2J2_SIAQTMSSEAQRPTTATQR.pdf]

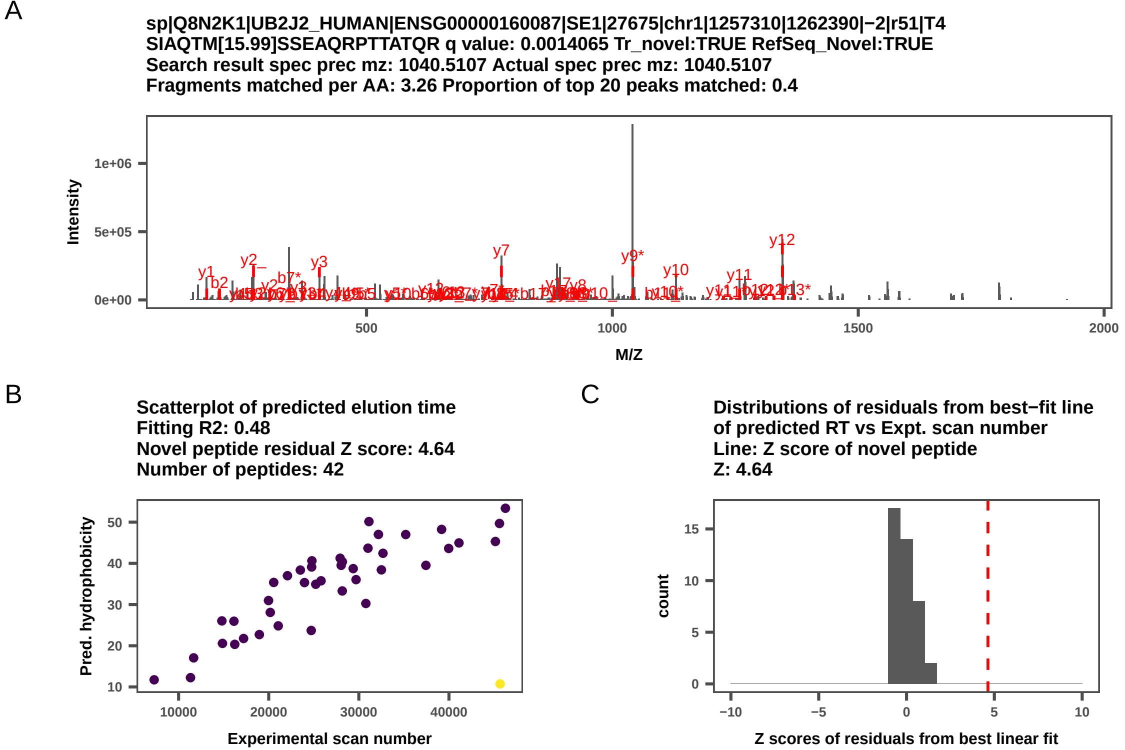
A
sp|Q8N2K1|UB2J2_HUMAN|ENSG00000160087|SE1|27675|chr1|1257310|1262390|−2|r51|T4
SIAQTM[15.99]SSEAQRPTTATQR q value: 0.0014065 Tr_novel:TRUE RefSeq_Novel:TRUE
Search result spec prec mz: 1040.5107 Actual spec prec mz: 1040.5107
Fragments matched per AA: 3.26 Proportion of top 20 peaks matched: 0.4
Intensity
1e+06
5e+05
0e+00
y1
b2
y2
b7*
y3
y7
y9*
y10
y11
y12
500
1000
1500
2000
M/Z
B
Scatterplot of predicted elution time
Fitting R2: 0.48
Novel peptide residual Z score: 4.64
Number of peptides: 42
Pred. hydrophobicity
50
40
30
20
10
10000
20000
30000
40000
Experimental scan number
C
Distributions of residuals from best−fit line
of predicted RT vs Expt. scan number
Line: Z score of novel peptide
Z: 4.64
count
15
10
5
0
−10
−5
0
5
10
Z scores of residuals from best linear fit
